# Sepsis as a confounding factor in assessing liver dysfunction in parenterally fed piglets: a model for human infants with intestinal failure

**DOI:** 10.1186/s42826-026-00279-5

**Published:** 2026-05-06

**Authors:** Mahabub Alam, Pamela R. Wizzard, Patrick N. Nation, Michael Zaugg, Paul W. Wales, Justine M. Turner

**Affiliations:** 1 Department of Pediatrics, University of Alberta, Edmonton, Canada; 2 Animal Pathology Services APS Ltd, Canmore Alberta, Canada; 3 Department of Anesthesiology and Pain Medicine, University of Alberta, Edmonton, Canada; 4 Department of Pharmacology, University of Alberta, Edmonton, Canada; 5 Department of Surgery, Cincinnati Children’s Hospital Medical Center and University of Cincinnati, Cincinnati, OH USA

**Keywords:** Total parenteral nutrition, Sepsis, Liver disease, Bile flow, Total bilirubin and piglet model

## Abstract

**Background:**

Sepsis is a common complication of parenteral nutrition therapy for infants with intestinal failure, due to the presence of a central venous catheter and impairment of gut barrier function. Intestinal failure-associated liver disease (IFALD) is another complication, often attributed to the type of intravenous lipid emulsion delivered and this is commonly studied in pre-clinical animal models. Animal studies frequently overlook sepsis as a confounding factor, potentially biasing the results. We retrospectively reviewed 14-day studies of total parenteral nutrition (TPN) in piglets in our laboratory from 2011 to 2025, to determine if sepsis is an independent predictor of key liver outcomes relevant to IFALD research. Sepsis was defined as positive blood culture.

**Results:**

A total of 86 piglets (76 male; aged 2–5 days) were eligible for inclusion across four experimental studies. In total, 44 (51%) were suspected to have sepsis on the basis of clinical signs, 4 had missing blood cultures, and of these 28/40 (70%) had a positive blood culture. Septic piglets had higher mortality compared to those not suspected to be septic or having a negative blood culture (43% vs. 9%, *p* < 0.001). Septic piglets showed significantly lower bile flow (*p* < 0.001) and higher serum total bilirubin (*p* < 0.001) compared to non-septic piglets. The risk of developing sepsis was reduced in piglets with older age (OR = 0.42, *p* = 0.007) and increased when given a pure soy-oil compared to pure fish-oil emulsion (OR = 10.77, *p* = 0.05). Multivariate analysis showed the independent predictors of bile flow were both sepsis (negative) and higher body weight at study entry (positive) (R^2^ = 0.42, *p* < 0.001), while sepsis and use of a soy-based lipid emulsion were independent positive predictors of total bilirubin (R^2^ = 0.34, *p* < 0.001).

**Conclusions:**

Sepsis is associated with reduced bile flow, elevated total bilirubin and increased mortality in PN fed neonatal piglets. As sepsis can be a confounding factor in liver outcomes, researchers should both introduce refinements to mitigate sepsis as well as consistently report on the number of animals with sepsis within treatment groups and their outcomes.

**Supplementary Information:**

The online version contains supplementary material available at 10.1186/s42826-026-00279-5.

## Background

Intestinal failure (IF) is the inability of the gut to absorb enough nutrients and fluids, requiring parenteral nutrition (PN) to sustain growth and development [1]. Although advancement of PN and multidisciplinary intestinal rehabilitation programs have significantly improved survival rates in infants with intestinal failure, prolonged PN dependency is associated with many complications. Intestinal failure-associated liver disease (IFALD) is an important complication of prolonged PN in infants that remains a leading cause of morbidity and mortality in this population [2]. Though IFALD is a multifactorial disease, soybean-based lipid emulsions and central line associated bloodstream infection (CLABSI) have been shown to be independent risk factors for IFALD in children [3]. Over time pediatric survival from IF has improved in part from decreased risk of IFALD due to advances in parenteral lipid emulsions [4]. In contrast, sepsis remains a major contributor to the development of IFALD in children with IF, contributing a 3-fold increased risk for IFALD, and significantly increasing mortality [3, 5–7].

Strategies to address IFALD have frequently been informed by studies in pre-clinical animal models, particularly the development and testing of new parenteral lipid emulsions [8–11]. However, preclinical studies often neglect to measure, report, or adjust for complications, which impacts the validity of findings and knowledge translation [12]. This includes sepsis and so may be a confounding factor for clinicians and scientists interpreting the results of preclinical TPN-IFALD studies. As well this may be a missed opportunity to explore the role of sepsis in IFALD pathogenesis, or to develop potential mitigation strategies for this important risk factor for IFALD. Our study objective was to retrospectively evaluate the impact of sepsis as a risk factor for key outcome measures of liver dysfunction in parenterally supported neonatal piglets. We hypothesized that sepsis will independently be associated with liver disease in PN-fed neonatal piglets undergoing experimental studies designed to evaluate the role of parenteral lipid emulsions in IFALD.

## Methods

We performed a retrospective review of neonatal piglets from our laboratory that were enrolled in 14-day total parenteral nutrition (TPN) trials, without enteral nutrition, between 2011 and 2025. We included experiments where comparisons were made of different lipid emulsion treatments or doses. Only treatments with a single commercial lipid emulsion for the 14 days were included. We excluded studies if they had any additional interventions, including vitamin E supplementation or trophic hormone therapy. The primary aim of this study was to determine if sepsis, defined as positive blood culture, was an independent predictor of key liver outcomes relevant to IFALD research in TPN fed piglets.

All included studies were similar in basic procedures. On study day 0, piglets underwent placement of a 5-French jugular central venous catheter under general anesthesia for continuous PN delivery [8, 13]. Perioperative prophylactic antibiotics were administered according to institutional protocols at the time of surgery. Parenteral nutrition commenced immediately following catheter placement and was advanced to full target volumes as previously described in our laboratory protocols [8]. In these studies piglets received either a pure soybean oil-based emulsion, pure fish oil-based emulsion, or a mixed oil emulsion, delivered at doses of either 5 g/kg/day or 10 g/kg/day [translates to the equivalent of 1 g/kg/day and 2 g/kg/day in a human infant, respectively]. Apart from lipid type or dose, PN composition was otherwise consistent across all studies.

Refinements in management for risk of sepsis occurred during the time period covered by this study. Before 2020, catheters were maintained with 1.5 mL heparin flushes twice-daily. For antibiotic prophylaxis, piglets received ampicillin (10 mg/kg twice a day; Sandoz, Boucherville, QB, Canada) and trimethoprim-sulfadoxine (100 mg/d; Merck Animal Health, Kirkland, QB, Canada) for four days postoperatively and again from days 8–12. After 2020, a 1 mL line lock using T-EDTA (4% tetrasodium ethylenediaminetetraacetic acid) was started from day 2 for 2 h daily. Antibiotic prophylaxis also changed to florfenicol (15 mg/kg IM, Intervet Canada Corp., Kirkland, QC, Canada) and ampicillin (10 mg/kg IV) postoperatively from days 0–4, with no additional antibiotic course routinely administered.

Piglets were housed individually in temperature-controlled metabolic cages and were monitored twice daily by trained staff for general health, catheter patency, and possible signs of sepsis including fever, lethargy and vomiting. Fever was defined as a rectal temperature > 39.5 °C, measured by a calibrated digital rectal thermometer. Lethargy was defined as reduced spontaneous movement, using a long-standing clinical scoring approach with visual prompts for laboratory staff to recognize changes in piglet behavior. Fever was interpreted along with other clinical signs including lethargy, vomiting, rather than considered a standalone sign of sepsis. These signs of possible sepsis triggered a treatment protocol that included obtaining a central line blood culture and commencing extra protocolized antibiotic treatment. Antibiotic treatments included enrofloxacin (5 mg/kg/d; Bayer Animal Health Mississauga, ON, Canada) and gentamicin (3 mg/kg/d; Sandoz, Boucherville, QB, Canada). Confirmed sepsis was defined as a positive blood culture obtained from the central venous catheter. If sepsis did not respond to antibiotic treatment then humane endpoints were applied.

### Liver outcome measures

On day 14, bile flow was measured via direct bile duct cannulation [8, 9]. Serum samples were collected and analyzed using automated biochemical methods (IDEXX Laboratories Inc., Edmonton, Canada) for total bilirubin, bile acids, gamma-glutamyl transferase (GGT) and alanine aminotransaminase (ALT).

### Statistical analysis

Comparisons were made using the t-test or Mann–Whitney U test for continuous variables, and the chi-square or Fisher exact test for categorical variables, as appropriate. Continuous variables were presented as mean ± standard deviation (SD) if normally distributed, or median (Inter quartile range, IQR) if non-parametric. Categorical variables were shown as frequency (%). To identify the predictors of sepsis, a univariate logistic regression was conducted with predictors including piglet breed, sex, baseline age, body weight at day 0 and day 14, type of lipid emulsion, lipid dose, and use of a line lock solution (T-EDTA). Following this, the univariate predictors significant at alpha ≤ 0.05 level were entered into backward stepwise multivariate logistic regression. To identify predictors of liver outcomes (bile flow, total bilirubin, bile acids, GGT and ALT) univariate linear regression was conducted with confirmed sepsis and the same predictors noted above, prior to backward stepwise multivariate linear regression including the significant univariate predictors (alpha ≤ 0.05). All analyses were performed using SPSS version 29 (IBM Corp, Armonk, NY, USA). Statistical significance was defined as *p* ≤ 0.05.

## Results

A total of 86 piglets were eligible for inclusion across four experimental studies. In total, 44 (51%) were presumed to have sepsis, 4 had missing blood cultures, and 28/40 (70%) had a positive blood culture. Thus, 82 piglets were included in the final analysis, distributed between the non-septic (*n* = 54) and septic (*n* = 28) cohorts. The characteristics of both cohorts are presented in Table 1. There were no significant differences in breed (*p* = 0.90), the majority of piglets studied were male (87.8%), with no apparent sex difference between non-septic and septic piglets (*p* = 0.15). At baseline, non-septic piglets were older (*p* = 0.009) and heavier (*p* = 0.005) than septic piglets. Similarly, by the end of the 14-day trials non-septic piglets weighed more (*p* = 0.004) and had gained more weight (*p* = 0.018) compared to septic piglets. Sepsis was more common in piglets that received a pure soybean oil-based lipid emulsion (59.4%) compared to pure fish oil (23.1%) or mixed oil (16.2%) (*p* < 0.001), while there was no difference in lipid dose between cohorts (*p* = 0.79). Piglets that received only heparin flushes had significantly higher sepsis rate than those that also received T-EDTA (40.6% vs. 11.1%, *p* = 0.02).

**Table 1:** Characteristics of non-sepsis and sepsis cohorts

Characteristics			Total Cohort (*n* = 82)	Non-Sepsis (*n* = 54)	Sepsis (*n* = 28)	*P* value
Breed, n (%)	LLW		49 (59.8)	32 (65.3)	17 (34.7)	0.90
	LLW×Duroc		33 (40.2)	22 (66.7)	11 (33.3)	
Sex, n (%)	Male		72 (87.8)	45 (62.5)	27 (37.5)	0.15*
Baseline age (days), mean (SD)			3.93 (0.99)	4.13 (0.99)	3.54 (0.88)	0.009
Day 0 weight (kg), mean (SD)			2.27 (0.24)	2.32 (0.22)	2.17 (0.26)	0.005
Day 14 weight (kg), mean (SD)			4.82 (0.57)	4.94 (0.50)	4.50 (0.64)	0.004
Weight gain (kg), mean (SD)			2.55 (0.43)	2.62 (0.39)	2.35 (0.49)	0.018
Lipid types, n (%) *		Pure fish oil	13 (15.9)	10 (76.9)	3 (23.1)	<0.001
		Mixed oil	37 (45.1)	31 (83.8)	3 (16.2)	
		Pure soybean oil	32 (39)	13 (40.6)	19 (59.4)	
Lipid dose, n (%)		5 g/kg/day	25 (30.5)	17 (68)	8 (32)	0.79
		10 g/kg/day	57 (69.5)	37 (64.9)	20 (35.1)	
T-EDTA, n (%)		Yes	18 (22)	16 (88.9)	2 (11.1)	0.02*

P values of categorical variables are determined by Pearson chi square (while p value by two-sided Fisher exact test designated by an asterisk *); and P values of continuous variables are determined by T test; SD, standard deviation

### Sepsis

Of the 28 piglets with positive blood cultures, gram-positive organisms were most frequent (79%), followed by 18% gram-negative organisms (18%) and 3% were mixed. The gram-positive organisms included *Enterococcus faecium* (*n* = 12), *Enterococcus faecalis* (*n* = 4), unspecified *Enterococcus spp.* (*n* = 1), *Staphylococcus epidermidis* (*n* = 2), *Staphylococcus simulans* (*n* = 1), *Staphylococcus hyicus* (*n* = 1), *Leuconostoc citreum* (*n* = 3), *Leuconostoc mesenteroides* (*n* = 1) and *Clostridium perfringens* (*n* = 1), either in combination or alone. The gram-negative organisms included *Klebsiella pneumoniae* (*n* = 3), *Enterobacter cloacae* (*n* = 1), *Escherichia coli* (*n* = 1), and *Bacteroides fragilis* (*n* = 1).

### Mortality

Septic piglets had higher mortality compared to those not presumed septic or having a negative blood culture (43% vs. 9%, p < 0.001) (see Table 2). Piglets with gram negative infections had higher mortality compared to piglets having gram positive infection, although this difference was not statistically significant (60% vs 41%, 0.63).

### Liver outcomes

**Fig. 1:**
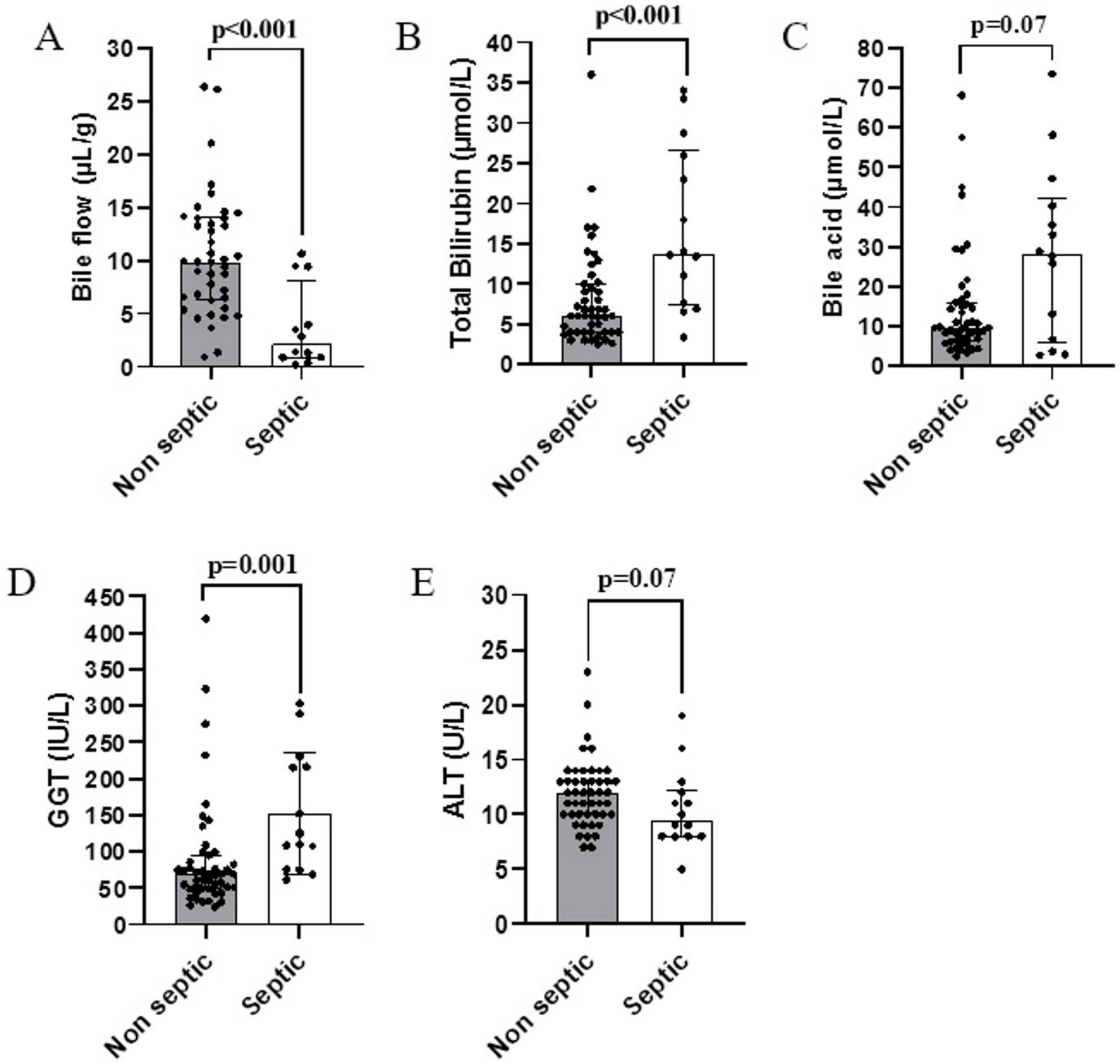
Comparison of liver outcomes between non-septic and septic cohort - (**A**) Bile flow (non-septic *n* = 41, septic *n* = 12); (**B**) Total bilirubin (non-septic *n* = 47, septic *n* = 14); (**C**) Bile acid (non-septic *n* = 47, septic *n* = 14); (**D**) Gamma-glutamyl transferase, GGT (non-septic *n* = 47, septic *n* = 14); (**E**) Alanine transaminase, ALT (non-septic *n* = 47, septic *n* = 14). P values were calculated by Mann–Whitney U test. Bars represent median and interquartile range. Each dot indicates individual sample per cohort

Septic piglets showed significantly lower bile flow (*p* < 0.001), along with higher serum total bilirubin (*p* < 0.001) and GGT (*p* = 0.001) compared to non-septic piglets (See Fig. 1). In contrast, serum bile acids (*p* = 0.07) and ALT (*p* = 0.07) did not differ between septic and non-septic piglets.

### Predictors of sepsis

Findings from the univariate analysis are shown in Table 3. The risk of developing sepsis was lower in piglets with older age (*p* = 0.013), having higher body weight at Day 0 (*p* = 0.008) and Day 14 (*p* = 0.007), or that received line lock solution, T-EDTA (*p* = 0.032). In contrast, the risk of sepsis was higher when the piglets received pure soybean oil-based lipid emulsions compared to pure fish oil (*p* = 0.035). Use of a mixed oil-based lipid emulsion was not associated with sepsis risk, compared to pure fish oil. Piglet breed, sex, and lipid dose were not significant predictors for sepsis. In multivariate analysis the risk of developing sepsis was reduced in piglets of older age (OR = 0.42, *P* = 0.007), and increased when given a pure soybean-oil compared to pure fish-oil emulsion (OR = 10.77, *p* = 0.05).

**Table 2:** Mortality outcomes

Group	Deaths	Mortality rate	*P* value
**Non-sepsis (n = 54)**	**5**	**9%**	Non-sepsis vs. sepsis (*p* < 0.001)
No suspicion of sepsis (*n* = 42)	2	5%	
Negative culture (*n* = 12)	3	25%	
**Sepsis (positive culture) (n = 28)**	**12**	**43%**	
Gram positive infection (*n* = 22)	9	41%	Gram positive vs. negative (*p* = 0.63)*
Gram negative infection (*n* = 5)	3	60%	
Gram (+/–) co-infection (*n* = 1)	0	0%	
**Total included**	**17**	**21%**	

P values correspond to group comparisons: non-sepsis vs. sepsis (Pearson chi-square) and Gram-positive vs. Gram-negative infection (Fisher’s exact test, *)

## Predictors of liver outcomes

Predictors of bile flow and total bilirubin are shown in Tables 4 and 5, respectively. While, the predictors of bile acids, GGT and ALT are listed in the Supplementary Tables 1, 2 and 3 respectively.

**Table 3:** Univariate and multivariable predictors of sepsis

Predictors		Univariate analysis		Multivariate analysis	
		OR (95% CI)	*P* Value	OR (95% CI)	*P* Value
Breed		0.94 (0.37–2.39)	0.90		
Sex		0.19 (0.02–1.54)	0.12		
Baseline age (days)		0.53 (0.32–0.87)	0.013	0.42 (0.23–0.80)	0.007
Day 0 weight (kg)		0.06 (0.01–0.48)	0.008		
Day 14 weight (kg)		0.24 (0.08–0.68)	0.007		
Lipid types	MO vs. FO	0.65 (0.14–3.07)	0.58	1.32 (0.12–14.55)	0.82
	SO vs. FO	4.87 (1.12–21.20)	0.035	10.77 (1.00-115.85)	0.05
Lipid dose		1.15 (0.42–3.13)	0.79		
T-EDTA		0.18 (0.04–0.86)	0.032		

Univariate predictors significant at ≤ 0.05 level were entered into backward stepwise multivariable logistic regression. OR, odds ratio; CI, confidence interval; FO, Pure fish oil; MO, Mixed oil; SO, Pure soybean oil

**Table 4:** Univariate and multivariable predictors of bile flow

Predictors	Univariate analysis			Multivariate analysis		
	**B**	**R²**	**P Value**	**B**	**R²**	**P Value**
Breed	-1.93	0.03	0.25		0.42	
Sex	0.74	0.002	0.74			
Baseline age (days)	2.15	0.14	0.006			
Day 0 weight (kg)	13.94	0.34	<0.001	11.33		<0.001
Day 14 weight (kg)	5.03	0.25	<0.001			
Lipid types	-4.20	0.17	0.002			
Lipid dose	-1.94	0.02	0.39			
T-EDTA	2.40	0.03	0.19			
Sepsis	-6.80	0.23	<0.001	-4.38		0.01

Univariate predictors significant at the ≤ 0.05 level were entered into backward stepwise multivariable linear regression. B, Unstandardized Coefficient; R²- Coefficient of Determination

**Table 5:** Univariate and multivariable predictors of total bilirubin

Predictors	Univariate analysis			Multivariate analysis		
	B	R²	P Value	B	R²	P Value
Breed	-1.30	0.01	0.54		0.34	
Sex	-4.19	0.03	0.15			
Baseline age (days)	-1.32	0.03	0.22			
Day 0 weight (kg)	-12.11	0.12	0.007	-6.94		0.09
Day 14 weight (kg)	-5.72	0.15	0.002			
Lipid types	5.81	0.21	<0.001	3.81		0.01
Lipid dose	3.75	0.04	0.11			
T-EDTA	-2.07	0.01	0.38			
Sepsis	9.02	0.22	<0.001	5.50		0.02

Univariate predictors significant at ≤ 0.05 level were entered into backward stepwise multivariable linear regression. B, Unstandardized Coefficient; R²- Coefficient of Determination

### Bile flow

In univariate analysis, we found that higher bile flow was associated with older age (*p* = 0.006) and higher body weight (at day 0 and day 14, p <0.001). While, the use of a pure soybean-oil based lipid emulsion (0.002) and sepsis (p <0.001) were associated with reduced bile flow. In multivariable analysis, both sepsis and lower piglet body weight at day 0 were independent predictors of reduced bile flow (R^2^ = 0.42, p <0.001).

### Total bilirubin

Univariate analysis showed that higher body weight at Day 0 (*p* = 0.007) and Day 14 (*p* = 0.002) were associated with lower total bilirubin. Use of pure soybean-oil lipid emulsion (*p* < 0.001) and sepsis (*p* < 0.001) were associated with higher total bilirubin. Our multivariable model showed that sepsis and use of a soybean-based lipid emulsion were independent predictors of elevated total bilirubin (R^2^ = 0.34, *p* < 0.001).

### Bile acids

Univariate analysis revealed that pure soybean-oil lipid emulsion (*p* = 0.016) and sepsis (*p* = 0.006) were associated with higher bile acids in serum, while, multivariate analysis showed that sepsis was an independent predictor of higher bile acids (R^2^ = 0.12, *p* = 0.006).

### GGT

In univariate analysis, we found that lower GGT level associated with older age (*p* = 0.018), higher body weight at day 0 (*p* = 0.005) and day 14 (p ˂0.001), and using T-EDTA for line lock (*p* = 0.048). While, the use of pure soy-oil emulsion (*p* = 0.04) and sepsis (*p* = 0.011) were associated with higher GGT level. Multivariable analysis showed that higher body weight at day 14 and use of T-EDTA were the independent predictors of lower GGT (R^2^ = 0.25, p <0.001).

### ALT

We found no significant predictors for serum ALT in our univariate analysis. Therefore, no variables were entered into multivariate analysis.

## Discussion

This preclinical cohort study in neonatal piglets in a single laboratory confirms that sepsis is not uncommon in TPN-fed piglets, can be associated with higher mortality and can be a potential confounding factor in preclinical research evaluating the role of parenteral lipids in IFALD. Sepsis was independently associated with two key liver outcomes, bile flow and total serum bilirubin. Interestingly, this parallels findings in human infants with intestinal failure [3]. In interpreting the results of preclinical studies for the assessment of IFALD it is important that clinicians understand the potential impact of sepsis (reported or unreported). Furthermore, the independent role of sepsis in IFALD warrants further pre-clinical and clinical research, to better understand pathogenesis and to potentially develop new mitigation strategies. Finally, developing such strategies (like use of T-EDTA) can reduce the risk of sepsis in preclinical animal research to improve animal welfare, reduce costs, and increase fidelity of research outcomes. Reporting sepsis as the potential confounder is an easy first step. On review of the published literature on TPN animal models to date it is clear that the incidence of sepsis is under-reported. Reviewing a total of 38 TPN animal studies that focused on liver disease as a primary outcome, we found only 21% of studies reported sepsis and only 47% reported animal mortality (see Supplementary Table 4).

Our septic piglets showed significantly lower bile flow along with higher serum total bilirubin and GGT compared to non-septic piglets. In clinical settings, IFALD is usually defined by persistent cholestasis and conjugated hyperbilirubinemia, which reflect impaired bile formation and excretion during PN dependency [1, 14]. The incidence of IFALD is more common in neonates with intestinal failure who get recurrent episodes of sepsis from CLABSI or bacterial translocation from the intestine. Sepsis possibly contributes to the development of IFALD through direct and indirect reduction in bile flow (interrupting enteral nutrition) and through the production of secondary bile salts [15]. Sepsis can directly decrease bile flow by upregulating hepatic production of proinflammatory cytokines (e.g., tumor necrosis factor-alpha, interleukin-1) and nitric oxide, which downregulate bile acid transporters [16, 17].

In our study, septic piglets most often grew gram positive organisms, while there was a trend to greater mortality when growing gram negative organisms. This observation is consistent with the human experience with CLABSI in children with intestinal failure, most commonly being due to gram-positive bacteria (62%) followed by gram negative bacteria (38%), and fungal infections (17%) [18]. Alterations of the gut–liver axis, including microbial dysbiosis of gut flora and associated inflammation, significantly contribute to IFALD pathogenesis, possibly due to the combination of the reduced bile flow, secondary bile salts production, and sepsis from bacterial translocation [15]. Refinements to our animal model, including changing our antibiotic protocols and use of T-EDTA for locking the central venous catheter both seem to have decreased the risk of sepsis over time [19]. Despite the added cost, we have previously reported that using 4% T-EDTA as an antimicrobial catheter lock reduces sepsis in a cost-effective manner in our animal research, consistent with emerging data in clinical studies [19–22].

In our piglet model older age and higher body weight reduced the risk of developing sepsis, which is plausible given that newborn piglets are very immature from an immunological point of view, receiving most of their immunoglobulins postnatally from sow milk [23]. Similarly, in human neonates, prematurity and low birthweight increase susceptibility to sepsis [18, 24]. Of note, use of pure soybean oil was associated with increased risk of sepsis. Soybean oil emulsions are rich in ω-6 fatty acids and phytosterols and exerts pro-inflammatory properties that could alter immune responses and increase susceptibility to bacterial infections leading to sepsis. However, to date it is controversial if there is a definite link between use of soybean-based lipid emulsions in increasing CLABSI, while data for fish oil lipid emulsions reducing CLABSI is lacking. Lenssen et al. [25] found no significant difference in bacteremia incidence between low- and standard-dose soybean-based lipid emulsions, while Pontes-Arruda et al. [26] demonstrated that lipid emulsions in premixed PN are not associated with increased infectious morbidity compared to lipid-free therapy. In contrast, restricting the dose of lipid emulsion to 1 g/kg/day in preterm infants with sepsis is associated with earlier resolution [27].

As a retrospective review, this study includes several inherent limitations. The number of female and T-EDTA animals was small, and the piglet breed changed over time with the addition of duroc genetics after 2012. Laboratory procedures to mitigate the risk of sepsis, including antibiotic protocols and use of T-EDTA did vary across time and different laboratory personnel were involved across study periods. All of these factors may have resulted in shifts in the microbiome in both the herd and the experimental animals over time that are relevant to IFALD as we noted, but were not measured in our studies. It is possible some septic piglets were misclassified as blood culture-negative, particularly given post-operative antibiotic exposure. Equally, although aseptic techniques were used, false-positive cultures can result from contamination during collection (although we did not see organisms typical of human skin flora). In addition, blood culture alone is not truly indicative of sepsis, and the measurement of acute phase proteins could help in refining our prediction mode by differentiating bacteremia from proven systemic sepsis. Despite these limitations, our findings demonstrate that sepsis acts as a potential confounder in preclinical research in PN fed neonatal piglets aiming to assess IFALD. We recommend careful monitoring and transparent reporting of sepsis in preclinical animal studies of this nature, as well as attention to mitigation studies to reduce the risk for sepsis and improve animal welfare, information that should be shared amongst this community of researchers.

## Conclusions

Sepsis is associated with reduced bile flow, elevated total bilirubin and GGT in PN fed neonatal piglets, as well as increasing mortality. As sepsis can be a potential confounding factor in key liver outcomes, researchers should introduce refinements that mitigate sepsis, and consider to exclude, or at least to report septic animals in all TPN studies. Finally, as sepsis is clearly an independent risk factor for IFALD it does warrant scientific evaluation in understanding IFALD pathogenesis, and as a target for prevention and treatment strategies to reduce IFALD. This should include better understanding in preclinical research of the role for lipid emulsions in increasing the risk of sepsis in TPN feeding.

## Supplementary Information

Below is the link to the electronic supplementary material.

Supplementary Material 1

## Data Availability

The data used and analyzed during this current study are available from the corresponding author on reasonable request.

## Abbreviations

ALT: Alanine transaminase
CLABSI: Central line associated bloodstream infection
GGT: Gamma-glutamyl transferase
IF: Intestinal failure
IFALD: Intestinal failure-associated liver disease
OR: Odd ratio
PN: Parenteral nutrition
R^2^: Coefficient of determination
T-EDTA: Tetrasodium ethylenediaminetetraacetic acid
TPN: Total parenteral nutrition
