# Supplementary material for: Sepsis as a confounding factor in assessing liver dysfunction in parenterally fed piglets: a model for human infants with intestinal failure
Source: Lab Anim Res. 2026 May 6;42:18. doi: 10.1186/s42826-026-00279-5 (PMC13147880; doi:10.1186/s42826-026-00279-5)
Supplement: Supplementary file 1 — Supplementary Material 1 [file 42826_2026_279_MOESM1_ESM.docx]

**Table 1: Univariate and multivariate predictors of bile acids**

| Predictors | Univariate analysis | | | Multivariate analysis | | |
| --- | --- | --- | --- | --- | --- | --- |
|  | **B** | **R²** | **P Value** | **B** | **R²** | **P Value** |
| Breed | -4.59 | 0.02 | 0.30 |  | 0.12 |  |
| Sex | -3.70 | 0.01 | 0.55 |  |  |  |
| Baseline age (days) | -2.65 | 0.02 | 0.24 |  |  |  |
| Day 0 weight (kg) | -14.44 | 0.04 | 0.13 |  |  |  |
| Day 14 weight (kg) | -6.40 | 0.04 | 0.10 |  |  |  |
| Lipid types | 8.10 | 0.09 | 0.016 |  |  |  |
| Lipid dose | -0.41 | 0.00 | 0.94 |  |  |  |
| ****T-EDTA**** | -0.67 | 0.00 | 0.89 |  |  |  |
| Sepsis | 13.89 | 0.12 | 0.006 | 13.89 |  | 0.006 |

Univariate predictors significant at ≤ 0.05 level were entered into backward stepwise multivariate linear regression. B, Unstandardized Coefficient; R²- Coefficient of Determination.

**Table 2: Univariate and multivariate predictors of GGT**

| Predictors | Univariate analysis | | | Multivariate analysis | | |
| --- | --- | --- | --- | --- | --- | --- |
|  | **B** | **R²** | **P Value** | **B** | **R²** | **P Value** |
| Breed | 9.39 | 0.003 | 0.67 |  | 0.25 |  |
| Sex | -53.39 | 0.05 | 0.08 |  |  |  |
| Baseline age (days) | -23.69 | 0.09 | 0.018 |  |  |  |
| Day 0 weight (kg) | -127.46 | 0.13 | 0.005 |  |  |  |
| Day 14 weight (kg) | -66.78 | 0.20 | ˂0.001 | -64.80 |  | ˂0.001 |
| Lipid types | 34.16 | 0.07 | 0.04 |  |  |  |
| Lipid dose | 22.18 | 0.01 | 0.36 |  |  |  |
| ****T-EDTA**** | -46.85 | 0.07 | 0.048 | -42.24 |  | 0.048 |
| Sepsis | 63.20 | 0.10 | 0.011 |  |  |  |

Univariate predictors significant at ≤ 0.05 level were entered into backward stepwise multivariate linear regression. B, Unstandardized Coefficient; R²- Coefficient of Determination.

**Table 3: Univariate and multivariate predictors of ALT**

| Predictors | Univariate analysis | | | Multivariate analysis | | |
| --- | --- | --- | --- | --- | --- | --- |
|  | **B** | **R²** | **P Value** | **B** | **R²** | **P Value** |
| Breed | 0.77 | 0.01 | 0.37 |  |  |  |
| Sex | 1.22 | 0.02 | 0.30 |  |  |  |
| Baseline age (days) | 0.67 | 0.04 | 0.12 |  |  |  |
| Day 0 weight (kg) | 2.61 | 0.03 | 0.16 |  |  |  |
| Day 14 weight (kg) | 1.12 | 0.04 | 0.14 |  |  |  |
| Lipid types | -0.31 | 0.004 | 0.64 |  |  |  |
| Lipid dose | -0.26 | 0.001 | 0.79 |  |  |  |
| ****T-EDTA**** | 1.010 | 0.02 | 0.28 |  |  |  |
| Sepsis | -1.46 | 0.04 | 0.14 |  |  |  |

No univariate predictors were not significant at ≤ 0.05 level to get entered into backward stepwise multivariate linear regression. B, Unstandardized Coefficient; R²- Coefficient of Determination.

**Table 4: Review on TPN animal models with central lines from 2005 to 2015**

| Author | Species | Intervention | Outcome | Sepsis | Mortality | References |
| --- | --- | --- | --- | --- | --- | --- |
| Lucchinetti et al. (2025) | Piglet | Lipid emulsions | Liver disease + others | Reported | Reported | [1] |
| Willis et al. (2025) | Mice | Lipid emulsions  (Cyclic vs continuous) | Liver disease | Not reported | Reported | [2] |
| Guthrie et al. (2025) | Piglet | Lipid emulsions | Liver disease | Not reported | Not reported | [3] |
| Manithody et al. (2024) | Piglet | TPN + Antibiotic + Intra duodenal fecal microbiota transplant | Liver disease + others | Not reported | Not reported | [4] |
| Cheng et al. (2024) | Rat | Liproxstatin-1 with TPN | Liver disease | Not reported | Not reported | [5] |
| Paulin et al. (2023) | Piglet | Lipid emulsions | Liver disease + others | Reported | Reported | [6] |
| Mims et al. (2023) | Mice | Lipid emulsions with antibiotic | Liver disease | Not reported | Reported | [7] |
| Yano et al. (2022) | Rat | Hepatocyte growth factor with TPN | Liver disease | Not reported | Not reported | [8] |
| Samaddar et al. (2022) | Piglet | TPN divided into low & high systemic inflammation. | Liver disease and microbiome | Not reported | Not reported | [9] |
| Guthrie et al. (2022) | Piglet | Lipid emulsions at various % | Liver disease | Not reported | Reported | [10] |
| Chen at al. (2022) | Piglet | Lipid emulsions | Liver disease + others | Not reported | Not reported | [11] |
| **Machigashira et al. (2021)** | Rat | Lipid emulsions | Liver disease | Not reported | Not reported | [12] |
| Call et al. (2020) | Piglet | Lipid emulsions | Liver disease | Suspected but no confirmation of sepsis | Reported | [13] |
| Guthrie et al. (2020) | Piglet | Lipid emulsions with Vit-E and Rifampicin | Liver disease | Not reported | Reported | [14] |
| Guzman et al. (2020) | Piglet | Lipid emulsions | Liver disease | Not reported | Not reported | [15] |
| Cao et al. (2020) | Rat | Lipid emulsions with exogenous secretin | Liver disease | Not reported | Reported | [16] |
| Lavallee et al. (2019) | Piglet | Lipid emulsions | Liver disease | Reported | Reported | [17] |
| Xu & Sun (2019) | Mice | Lipid emulsions at two doses | Liver disease | Not reported | Not reported | [18] |
| Yano et al. (2019) | Rat | Lipid emulsions with GLP-2 | Liver disease | Not reported | Not reported | [19] |
| Machigashira et al. (2018) | Rat | Lipid emulsions | Liver disease | Not reported | Not reported | [20] |
| Turner et al. (2016) | Piglet | Lipid emulsions | Platelet function | Reported | Reported | [21] |
| Lavallee et al. (2016) | Piglet | Surgical resection/remnant anatomy with SLE | Liver disease | Reported | Not reported | [22] |
| Guthrie et al. (2016) | Piglet | Lipid emulsions | Liver disease | Not reported | Reported | [23] |
| Onishi et al. (2016) | Rat | Lipid emulsions and ghrelin | Liver disease | Not reported | Not reported | [24] |
| Yang et al. (2016) | Dog | Lipid emulsions with different phytosterol level | Liver disease | Not reported | Reported | [25] |
| Josephson et al. (2015) | Piglet | Lipid emulsions at various doses | Liver disease + others | Reported | Reported | [26] |
| Turner et al. (2015) | Piglet | Lipid emulsions | Liver disease | Reported | Reported | [27] |
| Vegge et al. (2015) | Piglet | Lipid emulsions | Liver disease + others | Not reported | Reported | [28] |
| Vlaardingerbroek et al. (2014) | Piglet | Lipid emulsions | Liver disease | Not reported | Not reported | [29] |
| Jain et al. (2014) | Piglet | TPN with Ultramobile infusion pumps | Liver disease | Not reported | Reported | [30] |
| Kalish et al. (2013) | Mice | Lipid emulsions | Liver disease | Not reported | Not reported | [31] |
| Kalish et al. (2013) | Mice | Lipid emulsions | Liver disease | Not reported | Not reported | [32] |
| El Kasmi et al. (2013) | Mice | Lipid emulsions | Liver disease | Not reported | Not reported | [33] |
| Jain et al. (2012) | Piglet | Chenodeoxycholic acid (CDCA) and Lipid emulsions | Liver disease | Not reported | Not reported | [34] |
| Tazuke & Teitelbaum (2009) | Mice | Lipid emulsions | Liver disease | Not reported | Reported | [35] |
| Chen et al. (2009) | Dog | Lipid emulsions with Insulin ang glucagon | Liver disease plus others | Not reported | Not reported | [36] |
| Hyde et al. (2008) | Piglet | Lipid emulsions | Liver disease | Not reported | Reported | [37] |
| Alwayn et al. (2005) | Mice | Lipid emulsions | Liver disease | Reported no morbidity signs | Reported | [38] |

**References of Table 4**

1. Lucchinetti E, Lou PH, Chakravarty A, Marcolla CS, Pauline ML, Wizzard PR, et al. The Novel Lipid Emulsion Vegaven Is Well Tolerated and Elicits Distinct Biological Actions Compared with a Mixed-Oil Lipid Emulsion Containing Fish Oil: A Parenteral Nutrition Trial in Piglets. J Nutr. 2025;155(3):703–718.

2. Willis NB, Mims TS, Antunes K, Peng H, Yen MI, Yen CLE, et al. Cyclic infusion mitigates liver dysfunction associated with continuous total parenteral nutrition in a novel murine model. Am J Physiol Gastrointest Liver Physiol. 2025;329(4):G536–G545.

3. Guthrie G, Vonderohe C, Meléndez Hebib V, Stoll B, Burrin D. Multicomponent parenteral lipid emulsions do not prevent liver injury in neonatal pigs with obstructive cholestasis. JCI Insight. 2025;10(10).

4. Manithody C, Denton C, Mehta S, Carter J, Kurashima K, Bagwe A, et al. Intraduodenal fecal microbiota transplantation ameliorates gut atrophy and cholestasis in a novel parenteral nutrition piglet model. Am J Physiol Gastrointest Liver Physiol. 2024;327(5):G640–G654.

5. Cheng S, Wang Y, Zhao Y, Wang N, Yan J, Jiang L, et al. Targeting GPX4-mediated Ferroptosis Alleviates Liver Steatosis in a Rat Model of Total Parenteral Nutrition. J Pediatr Surg. 2024;59(5):981–991.

6. Pauline ML, Huynh C, Wizzard PR, Nation PN, Field CJ, Wales PW, et al. In parenteral nutrition-fed piglets, fatty acids vary by lipid emulsion and tissue sampled. J Parenter Enteral Nutr. 2023;47(8):1038–1046.

7. Mims TS, Kumari R, Leathem C, Antunes K, Joseph S, Yen MI, et al. Altered hepatic and intestinal homeostasis in a neonatal murine model of short-term total parenteral nutrition and antibiotics. Am J Physiol Gastrointest Liver Physiol. 2023;325(6): G556–G569.

8. Yano K, Sugita K, Muto M, Matsukubo M, Onishi S, Kedoin C, et al. The preventive effect of recombinant human hepatocyte growth factor for hepatic steatosis in a rat model of short bowel syndrome. J Pediatr Surg. 2022;57(7):1286–1292.

9. Samaddar A, van Nispen J, Armstrong A, Song E, Voigt M, Murali V, et al. Lower systemic inflammation is associated with gut firmicutes dominance and reduced liver injury in a novel ambulatory model of parenteral nutrition. Ann Med. 2022;54(1):1701–1713.

10. Guthrie G, Stoll B, Chacko S, Mohammad M, Style C, Verla M, et al. Depletion and enrichment of phytosterols in soybean oil lipid emulsions directly associate with serum markers of cholestasis in preterm parenteral nutrition-fed pigs. J Parenter Enteral Nutr. 2022;46(1):160–171.

11. Chen S, Xiao Y, Liu Y, Tian X, Wang W, Jiang L, et al. Fish oil-based lipid emulsion alleviates parenteral nutrition-associated liver diseases and intestinal injury in piglets. J Parenter Enteral Nutr. 2022;46(3):709–720.

12. Machigashira S, Kaji T, Onishi S, Yano K, Harumatsu T, Yamada K, et al. What is the optimal lipid emulsion for preventing intestinal failure-associated liver disease following parenteral feeding in a rat model of short-bowel syndrome? Pediatr Surg Int. 2021;37(2):247–256.

13. Call L, Molina T, Stoll B, Guthrie G, Chacko S, Plat J, et al. Parenteral lipids shape gut bile acid pools and microbiota profiles in the prevention of cholestasis in preterm pigs. J Lipid Res. 2020;61(7):1038–1051.

14. Guthrie G, Stoll B, Chacko S, Lauridsen C, Plat J, Burrin D. Rifampicin, not vitamin E, suppresses parenteral nutrition-associated liver disease development through the pregnane X receptor pathway in piglets. Am J Physiol Gastrointest Liver Physiol. 2020;318(1): G41–G52.

15. Guzman M, Manithody C, Krebs J, Denton C, Besmer S, Rajalakshmi P, et al. Impaired Gut-Systemic Signaling Drives Total Parenteral Nutrition-Associated Injury. Nutrients. 2020;12(5).

16. Cao X, Feng F, Liu X, Sun C, Yang X, Fang Y, et al. Exogenous Secretin Improves Parenteral Nutrition-associated Liver Disease in Rats. J Pediatr Gastroenterol Nutr. 2020;70(4):430–435.

17. Lavallee CM, Lim DW, Wizzard PR, Mazurak VC, Mi S, Curtis JM, et al. Impact of Clinical Use of Parenteral Lipid Emulsions on Bile Acid Metabolism and Composition in Neonatal Piglets. J Parenter Enteral Nutr. 2019;43(5):668–676.

18. Xu Z, Sun Y. The Role of Parenteral Lipids in the Development of Hepatic Dysfunction and Hepatic Steatosis in a Mouse Model of Total Parenteral Nutrition. J Nutr Sci Vitaminol (Tokyo). 2019;65(1):24–30.

19. Yano K, Kaji T, Onishi S, Machigashira S, Nagai T, Harumatsu T, et al. Novel effect of glucagon-like peptide-2 for hepatocellular injury in a parenterally fed rat model of short bowel syndrome. Pediatr Surg Int. 2019;35(12):1345–1351.

20. Machigashira S, Kaji T, Onishi S, Yamada W, Yano K, Yamada K, et al. The protective effect of fish oil lipid emulsions on intestinal failure-associated liver disease in a rat model of short-bowel syndrome. Pediatr Surg Int. 2018;34(2):203–209.

21. Turner JM, Field CJ, Goruk S, Wizzard P, Dicken BJ, Bruce A, et al. Platelet Arachidonic Acid Deficiency May Contribute to Abnormal Platelet Function During Parenteral Fish Oil Monotherapy in a Piglet Model. J Parenter Enteral Nutr. 2016;40(4):587–591.

22. Lavallee CM, Wizzard PR, Lansing M, Vine DF, Nation PN, Yap JY, et al. Surgical Anatomy Does Not Affect the Progression of Intestinal Failure-Associated Liver Disease in Neonatal Piglets. J Parenter Enteral Nutr. 2018;42(1):14–23.

23. Guthrie G, Kulkarni M, Vlaardingerbroek H, Stoll B, Ng K, Martin C, et al. Multi-omic profiles of hepatic metabolism in TPN-fed preterm pigs administered new generation lipid emulsions. J Lipid Res. 2016;57(9):1696–1711.

24. Onishi S, Kaji T, Yamada W, Nakame K, Moriguchi T, Sugita K, et al. The administration of ghrelin improved hepatocellular injury following parenteral feeding in a rat model of short bowel syndrome. Pediatr Surg Int. 2016;32(12):1165–1171.

25. Yang Z, Ren T, Lu D, Guo H, Li W, Huang C, et al. Evaluating the safety of phytosterols removed perilla seed oil-based lipid emulsion. Expert Opin Drug Deliv. 2016;13(10):1345–1356.

26. Josephson J, Turner JM, Field CJ, Wizzard PR, Nation PN, Sergi C, et al. Parenteral Soy Oil and Fish Oil Emulsions: Impact of Dose Restriction on Bile Flow and Brain Size of Parenteral Nutrition-Fed Neonatal Piglets. J Parenter Enteral Nutr. 2015;39(6):677–687.

27. Turner JM, Josephson J, Field CJ, Wizzard PR, Ball RO, Pencharz PB, et al. Liver Disease, Systemic Inflammation, and Growth Using a Mixed Parenteral Lipid Emulsion, Containing Soybean Oil, Fish Oil, and Medium Chain Triglycerides, Compared With Soybean Oil in Parenteral Nutrition-Fed Neonatal Piglets. J Parenter Enteral Nutr. 2016;40(7):973–981.

28. Vegge A, Thymann T, Lauritzen L, Bering SB, Wiinberg B, Sangild PT. Parenteral lipids and partial enteral nutrition affect hepatic lipid composition but have limited short term effects on formula-induced necrotizing enterocolitis in preterm piglets. Clin Nutr. 2015;34(2):219–228.

29. Vlaardingerbroek H, Ng K, Stoll B, Benight N, Chacko S, Kluijtmans LAJ, et al. New generation lipid emulsions prevent PNALD in chronic parenterally fed preterm pigs. J Lipid Res. 2014;55(3):466–477.

30. Jain AK, Wen JX, Arora S, Blomenkamp KS, Rodrigues J, Blaufuss TA, et al. Validating hyperbilirubinemia and gut mucosal atrophy with a novel ultramobile ambulatory total parenteral nutrition piglet model. Nutr Res. 2015;35(2):169–174.

31. Kalish BT, Le HD, Gura KM, Bistrian BR, Puder M. A metabolomic analysis of two intravenous lipid emulsions in a murine model. PLoS One. 2013;8(4).

32. Kalish BT, Le HD, Fitzgerald JM, Wang S, Seamon K, Gura KM, et al. Intravenous fish oil lipid emulsion promotes a shift toward anti-inflammatory proresolving lipid mediators. Am J Physiol Gastrointest Liver Physiol. 2013;305(11).

33. El Kasmi KC, Anderson AL, Devereaux MW, Vue PM, Zhang W, Setchell KDR, et al. Phytosterols promote liver injury and Kupffer cell activation in parenteral nutrition-associated liver disease. Sci Transl Med. 2013;5(206).

34. Jain AK, Stoll B, Burrin DG, Holst JJ, Moore DD. Enteral bile acid treatment improves parenteral nutrition-related liver disease and intestinal mucosal atrophy in neonatal pigs. Am J Physiol Gastrointest Liver Physiol. 2012;302(2):218–224.

35. Tazuke Y, Teitelbaum DH. Alteration of canalicular transporters in a mouse model of total parenteral nutrition. J Pediatr Gastroenterol Nutr. 2009;48(2):193–202.

36. Chen SS, Santomango TS, Williams PE, Lacy DB, McGuinness OP. Glucagon-mediated impairments in hepatic and peripheral tissue nutrient disposal are not aggravated by increased lipid availability. Am J Physiol Endocrinol Metab. 2009;296(5):1172–1178.

37. Hyde MJ, Amusquivar E, Laws J, Corson AM, Geering RR, Lean IJ, et al. Effects of lipid-supplemented total parenteral nutrition on fatty liver disease in a premature neonatal piglet model. Neonatology. 2008;93(2):77–86.

38. Alwayn IPJ, Gura K, Nosé V, Zausche B, Javid P, Garza J, et al. Omega-3 fatty acid supplementation prevents hepatic steatosis in a murine model of nonalcoholic fatty liver disease. Pediatr Res. 2005;57(3):445–452.
